# Japan should initiate the discussion on voluntary assisted dying legislation now

**DOI:** 10.1186/s12910-023-00886-0

**Published:** 2023-02-01

**Authors:** Atsushi Asai, Taketoshi Okita, Yoko Shimakura, Masashi Tanaka, Miki Fukuyama

**Affiliations:** 1 Department of Medical Ethics, Tohoku University Graduate School of Medicine, School of Public Health, 2-1 Seiryo, Aoba-Ku, Sendai, Miyagi 980-8575 Japan; 2 KARADA Internal Medicine Clinic, Tokyo, Japan; 3 Division of Clinical Epidemiology, National Hospital Organization Tokyo Medical Center, Tokyo, Japan; 4 Course of Nursing, Faculty of Life Sciences, Kumamoto University, Kumamoto City, Japan

**Keywords:** Voluntary assisted dying (VAD), Voluntary Assisted Death Act 2017 (Victoria), Safeguards, Japan, Culture, Objections, Discussion

## Abstract

**Background:**

No laws or official guidelines govern voluntary assisted dying (VAD) in Japan. A legislative bill on the termination of life-sustaining measures has yet to be sent to deliberations for legislation, due to strong opposition that has prevented it from being submitted to the Diet. However, Japan has recently witnessed several cases involving VAD.

**Main text:**

Against this backdrop, we argue that Japan should begin discussion on VAD legislation, referring to the Voluntary Assisted Dying Act 2017 (VADA2017), which was established in 2017 in Victoria, Australia. VADA2017 puts in place a wide range of stringent safeguards and is considered worldwide to be the safest and most conservative policy on a physician offering assisted dying based on the patient’s premeditated request. We consider what opposing opinions from society would arise in response to the VADA2017. Among these will include arguments against VAD itself, those against the validation of this act, and opinions that oppose even the initiation of the dialogue on VAD.

**Conclusions:**

We conclude that to protect the right to life among those placed in vulnerable positions and, at the same time, to respect decision-making of those who wish for immediate death due to unbearable suffering, the dialogue must immediately begin with that on introducing a policy more conservative than that of the VADA2017, which solidly considers arguments against VAD.

## Background

Legalization of voluntary assisted dying (VAD) has been progressing in several countries across the world [1–5]. In these countries, the individual’s right to self-determination with regard to their own death is affirmed under certain conditions, and some may even say that it is socially established. Meanwhile in Japan, a bill that forms the basis of a law may be submitted to the Diet for consideration by a member of the Diet or the Cabinet. The bill is debated in both the House of Representatives and the House of Councillors, and becomes law when both houses unanimously vote that the bill is appropriate as a new law. However, a legislative bill on the termination of life-sustaining measures has yet to be sent to deliberations for legislation at the Diet due to strong opposition that has prevented it from debating in either the House of Representatives or the House of Councillors at the Diet [6–8]. In addition, according to Japan’s penal code, “solicitation of suicide (the act of intentionally killing oneself),” “assistance in suicide,” “commissioned murder,” and “consensual homicide” are all illegal [9]. There have been no special legal regulations concerning VAD in Japan involving physicians. Moreover, very little discussion has occurred with regard to the matter of dying with dignity [10].

In Japan, the famous judicial precedent regarding assisted dying is the Tokai University Hospital Case in 1991, in which a physician administered potassium chloride to a patient according to the demands of the patient’s family, resulting in the patient’s death [6, 11]. On March 28, 1995, the Chief justice of the Yokohama District Court described four new legal requirements for “physician-assisted voluntary euthanasia” (precipitating the advent of death of a competent patient who is suffering uncontrollably and explicitly wishes to terminate his or her life by direct interferences by the physician [12]: (1) The patient must be suffering from unbearable physical suffering; (2) the patient’s death must be unavoidable and imminent; (3) every possible palliative treatment and care to ease the patient’s physical suffering must have been provided, and no alternatives must be available; and, (4) the patient must have expressed a clear and voluntary desire to have his or her life shortened [11]. However, since Japanese court decisions are based neither on precedents nor antecedents, and because these requirements were ruled at such at an incidental level at one regional court, they carry no official legal significance.

In recent years, Japan has witnessed an increasing number of cases involving VAD, despite the relative rarity of such cases historically. From 2018 to 2019, there were three cases in which the patient premeditatively requested assisted dying and arguments emerged in Japan concerning the right to self-determination of one’s own death by patients [5, 9, 13–16]. The common link between these three cases is that there was a clear and premeditated request for death from the patient. Moreover, until the moment before death, the patient was in a fully conscious state, the terminal stage was not necessarily imminent, there was physical and emotional suffering from which no recovery was possible, death would have been difficult to accomplish on their own due to physical disabilities, and the necessary medical care and caregiving were being provided [13]. Data from multiple reports and an assisted dying organization from Switzerland have clarified that Japanese citizens and residents have undergone assisted dying in Switzerland [17].

Against this backdrop, the present paper argues that Japan should immediately begin discussion on VAD legislation, referring to the Voluntary Assisted Dying Act 2017 (hereafter, VADA2017), which was established in 2017 in Victoria, Australia. VADA2017 puts in place a wide range of stringent safeguards and is considered worldwide to be the safest and most conservative policy on a physician offering assisted dying based on the patient’s premeditated request and end-of-life matters [18, 19]. The reason we chose to examine the VADA2017 for the present paper was that it is the safest and most conservative legislative policy on VAD, and we expect that it would cause the least amount of resistance if Japan entered into discussion about its incorporation. More specific reasons are given in the next section.

Below, we first introduce the VADA2017 and its characteristics. Following this, we present the opposing arguments that may be brought forth against our proposal to begin dialogue on the validation of VAD. Among these will include arguments against VAD itself, those against the validation of this act, and opinions that oppose even the initiation of the dialogue on VAD. We conclude from these discussions that, in order to protect the right to life among those placed in vulnerable positions by various definitions and, at the same time, to respect decision-making of those who wish for immediate death due to unbearable suffering, the dialogue must immediately begin with that on introducing a policy even more conservative than that of the VADA2017, which solidly considers arguments against VAD.

## Main text

### Characteristics of the Voluntary Assisted Dying Act 2017 (Victoria)

The VADA2017 is one of the safest and most conservative of all existing social policies on VAD [18, 19]. It has strict eligibility criteria and documentation, penal provisions, mandatory reporting, and as many as 68 safeguards built into it [18–21]. The VADA2017 clearly states guiding principles including equal value of every human life; respect for a person’s autonomy; supported informed decision-making and conversations about treatment and care preferences in a stable therapeutic relationship; quality care that minimises suffering and maximises quality of life, open discussions about death and dying; genuine choice balanced with safeguards protecting individuals who may be subject to abuse; and the right of all people to be respected for their culture, beliefs, values, and personal characteristics [22]. Characteristics of the VADA2017 are as follows [18, 19, 21, 23–27]:

1. To be eligible, a person must be 18 years or older with decision-making capacity, and have an incurable, advanced, and progressive disease that leaves them with a prognosis of six months or less to live (or one year in the case of a patient with neurodegenerative disease), and someone whose disease is generating suffering that cannot be relieved in a manner that the person deems tolerable.
2. In order to access assisted dying, a person must have made a formal request on three occasions, each of which must be separated by 9 or more days. The physician must not initiate the discussion about VAD; if a colleague is found to be doing this, the relevant agency must be notified. Only the person deciding to seek VAD (not his or her caregiver, family, friend, or support person) can ask for it.
3. A person must undergo evaluations by two independent physicians to assess their eligibility. Any physicians assigned to evaluate eligibility must have undergone training on VAD. Physicians are not obligated to participate in VAD and their right to conscientious objection is respected.
4. The default would be for a person to self-administer the medication required for VAD.
5. At each stage of the process, mandatory reporting is enforced and a system is established to ensure review monitoring.
6. A prospective approval and oversight process is used and a governmental permit is needed.

Bullet points 5 and 6 make the VADA2017 different from international regimes, as well as more conservative than others. The Department of Health in Victoria also states that VAD is not an alternative to palliative care services [27].

Specific reasons we chose to examine the VADA2017 for the present paper are as follows: 1) the VADA2017 clearly states that the physician must not initiate the discussion about VAD. This rule prevents patients from wishing for VAD according to their physician’s suggestion or pressure. 2) The default administration of VAD medication would be for a person to self-administer the medication required for VAD. This rule could prevent the case where lethal drugs are administered by a physician while the patient's intention is ambiguous and, as a result, guarantees the patient's voluntary action. 3) Only the person deciding to seek VAD, and not his or her caregiver, family, friend, or support person, can ask for it. This rule prevents the occurrence of VAD in cases for which the patient applies for it due to outside pressure. 4) A prospective approval and oversight process is rigorously used and a governmental permit is needed. This rule is very important because physicians feel more comfortable proceeding with the VAD procedure if they receive official permission before doing so. This is because Japan currently lacks written legal regulations concerning the termination of medical intervention, and healthcare professionals are left with uncertainty about which actions are forbidden. A patient’s right to refuse life-sustaining treatment (including withholding and withdrawal) has not been substantially warranted, and advance directives are still not legally enforceable, even today [28]. Moreover, in East Asian medical practice, where physicians are increasingly wary of possible lawsuits, there is an increasing dependency on clearly defined legal documents [29]. Therefore, in the absence of legal protection, few physicians dare to help patients seeking VAD [30].

### Possible objections to the commencement of discussion of voluntary assisted dying legislation in present Japan

The main content that has been controversial with regard to VAD in Japan is fundamentally very similar to that in global arguments surrounding the VAD debate. Those in favor of VAD argue for the right to choose, desire for autonomy, control of one’s own life, relief from suffering, the benefit of releasing a patient from their suffering, and dying with dignity. Meanwhile, the opposition cites the potential for abuse from which a slippery slope could develop, the potential coercion of vulnerable people by family and society, physician error, violation of the objectives of medicine, religious objections, and moral opposition, to name a few [31, 32]. Arima advocates that there is value in one’s existence alone, that this value should be prioritized over an individual’s self-determination or benefit, and that VAD would infringe on the intrinsic value of human life [16]. Against the VADA2017 in Victoria, the following objections have been reported: palliative care is effective; the purpose of medicine does not include assisting in death; there is a substantive risk of the socially vulnerable being driven to death involuntarily; and no one has the right to kill another person [18].

Below we mention some objections to VAD that seem to be more prominent in Japanese society than in other cultures. At the basis of these objections are cultural characteristics related to decision-making and human relationships in Japan as well as the countries that share cultural tendencies. These cultural inclinations include an emphasis on family-centered decision-making and filial piety; an emphasis on the importance of interpersonal relationships and harmony; the importance of a spirit of caring for and worrying about the emotional reactions of others; respect for, or obedience to, authority and other-oriented tendency; strong closeness, a homogeneity norm, and the undifferentiated nature of the individual (i.e., no clear differentiation between oneself and others) [6, 8, 12, 30, 33]. Here, we examine some oppositional thoughts that are most likely to emerge in this case. These have been divided into three groups.

#### Opposition and skepticism against voluntary decision-making

##### Death is not an issue involving just the individual requesting VAD

Some would express the opinion that, while the individual’s intent is certainly a necessary condition, it is not the only condition required; rather, it represents only the starting point of the argument. A human life, which of course includes their death, is not only theirs, but rather shared deeply and symbiotically by those around them as well; as such, it is not a matter that can be resolved by self-determination alone. In cases for which the family is opposed to VAD, it is argued that it should not be allowed [1, 9, 34]. The document that most explicitly expresses this is the 2020 Guideline on Dialysis-related Self-determination, which states, “At the point when dialysis becomes required, if the patient does not elect renal replacement therapy, then conversations should be had repeatedly between the patient and their family/heirs until a consensus is formed [35].”

##### The person turns to VAD out of voluntary consideration toward others

There have been frequent discussions on the strong influence of psychological, cultural, and social trends regarding human relationships on an individual’s decision-making within the Japanese society and this likely affects the individual’s self-determination concerning death [33]. These include ‘surmise (*sontaku*),’ ‘self-restraint *(jishuku*),’ ‘air (atmosphere or mood, *kuuki*),’ and ‘peer pressure (or tuning pressure, *docho-atsuryoku*).’ All of these, often referred to as characteristics of present-day Japanese people, may affect personal decision-making in clinical settings [33].

‘Surmise’ means reading the ‘air’ (atmosphere), recognizing the superior’s intentions in advance, and deciding to act accordingly. Some claim that ‘surmise’ is based on one’s consideration for others, as the Japanese are always semi-consciously taking note of other people’s moods and feelings [36]. ‘Self-restraint,’ or self-regulation, is the voluntary refraining from doing things when one wishes to do them [37]. It is argued that Japanese people would quietly and gently refrain from engaging in a certain behavior even if refraining is optional and lacks coercion or penalties, simply as a result of their practice of ‘reading the air’ [38].

Many Japanese people likely view themselves not only as an individual life, but as one deeply rooted in interrelationships with family and others as well. These trends may be influencing an individual’s thoughts regarding VAD. For example, having considered the thoughts and feelings of the head of the household or breadwinner, or those in charge of the patient care, some may come to desire VAD out of ‘surmise’ for others. Death may also come about as a result of a patient ‘self-refraining’ from life-sustaining measures as they try to ensure that they do not become a care burden or financial burden on their families.

##### Someone may desire VAD out of peer pressure caused by a discriminatory environment (i.e., non-voluntary assisted dying)

In Japanese culture, where individualism is not emphasized, the group mentality and peer pressure are stronger driving factors, and even those with differing opinions are expected to follow along obediently with what the group decides; some have argued that an individual’s strength to resist peer pressure is quite weak [9, 39]. As pointed out by Japanese psychiatrist Wada, in relation to the COVID-19 pandemic, Japanese ‘peer pressure’ is so strong that it prevented the expression of individual opinions out of fear of criticism; moreover, there is no true appreciation of the importance of freedom, and people gently obey the government policies, while also believing what the mass media reports without questioning [40]. Accordingly, there is some concern that patients with severe illnesses or disabilities may respond to discriminatory social ‘air’ or peer pressure implying that the patient and society would be better off if the patient was dead, arriving at a desire for their own death [39]. Others have argued that if VAD is legalized, Japanese society will face a high risk of patients being murdered due to peer pressure [41].

‘Peer pressure’ (*docho-atsuryoku*) refers to the power that implicitly forces a minority or dissident to act like a majority; in other words, this is pressure enforcing the notion that “everyone must be the same,” an order to read and obey the ‘air’ of a majority or mainstream group [38]. In Japan, people tend to be accused of both making individual decisions and taking unique actions, and it is argued that brainwashing is being carried out in the field of education, establishing firmly in the mind of children that obedience is a good thing [42]. In a recent paper, Asaguro refers to the risk of public opinion by noting that, although everyday selfless acts to achieve social harmony are mostly harmless, there is a risk of patients choosing death to bring about a harmonious whole [8]. Particularly worrisome may be the general public’s opinion, which could have a grave influence on individuals [43]. In this context, this general public’s opinion can be regarded as the ‘air.’

##### The person tends to avoid self-determination

Nakazawa et al. argue that Japanese culture places some value on the practice of not making decisions. In Japan, individualism is overshadowed more by traditional culture. In medical settings, patients tend to lean more toward no decision-making of their own regarding the course of their treatment, electing instead to have someone else decide what is best for them [6]. The desire concerning the decision-making about one’s subjective, voluntary, and clear request for death is the main premise behind VAD. If someone who is trying to bypass self-determination and wishing for someone else to take over the decision-making comes to utilize the VAD system, what should be a voluntary process might ultimately become non-voluntary, as pointed out in the slippery slope argument below.

##### A physician’s discriminatory empathy could lead to a person’s death

Arima and Matsuda have opined that cases might emerge in which a patient is led to death as a result of a medical caregiver’s ‘empathy’ [16, 44]. There is a fear that various prejudices may be hidden within a medical caregiver’s ‘empathy’ that living in this condition would be very difficult. For example, the caregiver may think, “It would be difficult to be bedridden with amyotrophic lateral sclerosis (ALS),” but the ‘empathy’ might reflect a negative sense of value toward ALS and other functional disorders. There is also a concern that elderly individuals, those with low-income status, and those without family would be susceptible to the ‘empathy’ reflecting a negative sense of value, i.e., a discriminatory feeling. If a physician responds with ‘empathy’ to the patient’s lack of support or financial difficulties, the physician might turn more readily toward practicing VAD for these patients. This would arguably be spurred on even further if VAD was legalized. The legalization would place a higher risk of death on those within society who “don’t look happy” [45, 46]. It is also pointed out that patients deemed unworthy of living might be viewed also as those unworthy of receiving medical care resources [47]. A commentator has strongly opposed the notion that there is no value in living if one has lost the purpose of living (‘*ikigai*’), or if one can no longer contribute to society [47].

#### Oppositions and fear toward the legalization of voluntary assisted dying (VAD)

##### Individuals blindly follow the law

In Japanese society, there has been some hesitation toward legalizing VAD and opening this up to the public. There is a problem with how the law is embraced by Japanese society, which is said to be because Japanese citizens tend to assume a stance of “once something is decided for the public, we must strive to follow this as much as possible.” If VAD were to be legalized, the public scrutiny that could potentially imply “why aren’t you dying/why aren’t you allowing them to die?” may inevitably be directed at patients or families who find themselves in situations in which VAD might be an option, but may not be representative of the patient’s desire to hold on to their life until a natural death occurs [9].

##### The slippery slope argument

Slippery slope arguments typically claim that something terrible will happen if we did a certain arguably desirable thing [48]. With the legalization of VAD, there are concerns that some physicians will become reckless with their patients’ capacity to live, or abuse the law and not report the correct cause of death [1]. Matsuda argues that, historically, a clear delineation between VAD and non-VAD has not always been present [44], and that there exists no clear psychological distinction between the former and the latter using the following examples: In the first scenario, Person A commits suicide, thinking, “If I’m in this much suffering, I’d rather just die quickly.” In the second scenario, Person A witnesses that B, who is close to A, is in so much suffering and begs A to help B to die. Person A, unable to see B suffering, feels compassion for B and lends a hand in B’s dying. Emotionally, the distance between the two scenarios is not that significant. In addition, the patients may have not clearly expressed their wishes, but if they are in agony and an onlooker feels that life would not be worth living if it is in this manner, then the patients’ death may be elected by the onlooker. That person may have reasoned that the patient would certainly wish for death. Thus, taking one step beyond VAD, i.e., that toward non-VAD, would not be too unnatural. Therefore, it is claimed that VAD is not necessarily clearly distinguishable from non-VAD [44].

Incidences of euthanasia (precipitating the advent of death of a patient who is suffering uncontrollably by direct interferences by the physician) in the 1990s in Japan were committed by physicians against terminal or comatose patients, all cases lacked patient intention regarding their desire to die, and yet death was administered according to the judgment of family members and attending physicians [11]. It is certainly possible to argue that we should not make a black and white decision about VAD legislation out of concern for the law taking off on its own, and that clearly stipulating it in the law makes civilian control more difficult.

#### Objection to beginning discussions on VAD legislation based on the reasoning that the timing is premature

The patient’s right to self-determination should obviously be respected and valued. However, shared acknowledgement within Japanese society about whether or not that should include the patient’s right to die (i.e., VAD) has not yet been cultivated. Some have therefore advocated that it is still too early to have a debate on its legalization [49]. One argument states that the first step is to open the discussion about upgrading and enhancing medical care and caregiving systems to ensure that these individuals feel fully supported in their right to life. Proponents of this argument would say that allowing hasty discussions to direct these important decisions about VAD unforgivable offense, and that the discussion on the right to life must come first [49].

Similarly, in a society with inadequate palliative care options, some have argued that it is too early to begin discussions on the legalization of VAD [50]. In addition, as noted above, there are no laws in Japan that address the patient’s rights or medical care decisions in terminal stages, and no rules or regulations even exist on withholding or withdrawing treatment. Given those circumstances, some might argue that suddenly beginning the debate about a law on VAD is premature, and that it should begin instead with a discussion on drafting legislature pertaining to withholding or withdrawing treatment. For Japanese society, even beginning the debate on VAD is in itself perhaps impermissible, as some are likely concerned that people will start to talk more easily about death, come to view death with less gravity, and consider VAD more readily, and that more anxiety will develop in patients with severe illnesses or disabilities, along with other negative effects such as a distrust in medical care.

Before concluding this section, we would like to present one additional note on Japanese culture. Some of the above views argue against the implementation of VADs, pointing out that Japanese psycho-socio-cultural tendencies, interpersonal relationships, the burden on the patient’s family, and the one-sided values of physicians may prevent appropriate VAD implementation and undermine assisted dying that is based on the true will of the individual. They attempt to oppose the implementation of VADs, by pointing to their potential to do so in the worst scenario. However, the intense human relationships in Japanese culture could also give members of society a stable sense of belonging, a sense of social role, solidarity, meaning of life, and purpose in remaining alive. They also provide friendly and kind care to the sick and allow ill individuals to rely casually on others. These characteristics may give patients the desire to persist in their end-of-life, no matter what their conditions are. In addition to familism, harmony, and other-oriented tendencies, Japanese culture embodies an intense supremacy of life or sanctity of life rooted in Shintoism and Confucianism. Death is regarded as the ultimate impurity and considered a serious taboo, and any discussion of death is still highly discouraged and met with great resistance. Patient refusal of life-prolonging treatment, especially the withdrawal of such, is often not accepted by the physician or family. Furthermore, there is no definite concept of an afterlife. Therefore, it should be noted that in many cases, doctors, families, and society still prefer to keep patients alive, regardless of their wishes or suffering [28, 30, 51]; this existence of this cultural tendency is precisely why we argue that we should begin to discuss the VAD in Japanese society.

## We should begin to discuss the acceptability of a VAD act which is safer and more conservative than the Voluntary Assisted Dying Act 2017 (Victoria)

Some of the authors have challenged a number of the above-mentioned views opposing VAD, pointing out in detail the ethical and philosophical problems inherent in the objections elsewhere [13]. Nevertheless, we believe that all of these views need to be systematically addressed if a VAD procedure is to be incorporated into Japan’s social and legal system from a practical point of view.

Nevertheless, we also feel it inappropriate to conclude that, because of the various concerns about the issue, the discussion about VAD legislation is premature or unnecessary, and therefore should not be initiated. There are some reasons for this. First, in the current state, illegal incidents are already underway, as individuals in Japan are going abroad to undergo VAD; these cannot be ignored [5, 9, 14, 15, 52, 53]. We must not forget that there are those living in suffering right now [13].

Second, we must not ignore recent opinions of Japanese citizens on the topic of VAD. Some Japanese people are thought to desire active euthanasia (precipitating the advent of death of a competent patient who is suffering uncontrollably and explicitly wishes to terminate his or her life by direct interferences by the physician); at the very least, 1 in 20 are estimated to think this way [9, 31, 54]. Groenewoud et al. suggest in their latest study that, asked for their preferred medical decision at the end of life if they would become terminally ill, 18.0% of the Japanese surveyed prefer the active ending of life [55].

Third, postponing or simply not having the discussion on this matter will make it difficult to discover or create common ground. Sasaki states that the recent commissioned murder case of ALS patients in Japan is a tragedy occurring in Japanese society because Japan continues to postpone or even fails to rule on the Death with Dignity Act [56]. In addition, it is rare that 100% of people can completely approve any issue; in fact, one might argue that it is precisely because of the impossibility of consensus formation on normative items that each individual’s self-determination and freedom must be viewed as ever more important.

Fourth, it is possible that one common opposing argument we hear against the VAD legislation as taken up in Japanese society today does not necessarily represent the opinions of the entire Japanese population. It may be easier for opposing voices to be taken up more readily by the mass media [57–59]. A public discussion in which affirmative and opposing opinions are calmly heard and balanced is needed. Finally, a direct and honest discussion must be initiated, and mutual trust must be cultivated by exchanging opinions. If mutual understanding is lacking in the process of discussion, then no amount of discussion will give rise to trust.

## Conclusions

As Weston suggests, both life and choice matter, and all of us value both life and choice. Freedom from pain matters, autonomy matters, and respect for life matters. Many people on all sides of this issue have been willing to accept a policy that allows VAD in some cases under tightly controlled conditions. Essentially it is a compromise [60].

In the present paper, we advocate that Japan should initiate the discussion on introducing a system even more conservative than the VADA2017 to address the current situation in Japan and the concerns of those opposed to VAD, in order to ensure that the right to life among individuals placed in various vulnerable positions, as well as the freedom of those desiring death due to unbearable suffering, can coexist within Japanese society. In addition to the guiding principles of the VADA2017, we suggest that the following principles will be necessary.

First, each individual must be valued, and acknowledging individuality will be important. It will also be valuable to ensure that no form of pressure is exerted toward another person, including those in one’s own family. Even within families, conflicts of interest and differences in values can develop, and given that there are also cases of failed family relationships, VAD with the unconditional premise that a family consensus is required is not desirable. That said, it will obviously be important to show consideration toward the family members of those who elect VAD. Therefore, in addition to establishing opportunities to confirm each person’s intents individually on multiple occasions, promoting family participation in the decision-making process for VAD will likely be necessary.

Second, by articulating the objectives and basic principles of the legalization, and by establishing safeguards even more stringent than those of the VADA2017 that would prevent negative outcomes due to negative influences from Japanese culture, we believe that it will be critical to continue to affirm that under no circumstances is anyone “obligated to die.” Sasaki clarifies that VAD is an act that one voluntarily ‘does’ and not something that is ‘done’ to a person [56]. It is always important to recall the moral basis of permitting VAD: at its core, it is about respecting and supporting the self-regarding choices made by patients with decision-making capabilities when they suffer from a grievous and irremediable medical condition that causes their suffering to be intolerable [58]. Japan will require all of the VADA2017 safeguards, including a prospective approval and oversight process, self-administration default, and regular monitoring. Additionally, in Japanese culture and government policies, various strategic measures will need to be implemented to ensure that rights concerning an individual’s death never transform into obligations. The first VAD law to be proposed in Japan should be very conservative. We would like to propose the addition or modification of the VADA2017, with the assumption that after a certain period of time, it will be carefully modified. First, the individual should always be examined by a psychiatrist as a third doctor to check thoroughly whether the individual is not under external pressure, whether the intention is truly voluntary, and furthermore, to rule out any psychological problems. Second, we must eradicate any desire for death based on peer pressure, which can come from a vague and terrifying atmosphere that may seem to resemble empathy. To that end, education of patients and all personnel involved in Japan’s predominantly emotional, cultural, and societal trends is even more important than that in other cultural regions [33]. There is also a need to offer education related to VAD as it pertains to healthcare specialists, as well as education to eliminate inherent prejudices. Arbitrary and immediate attempts at bedside resource distribution by individual physicians aiming to reduce medical spending should be prohibited. Third, any legislation must clarify that reduced medical spending is not one of the objectives of VAD legislation. It should also be clarified that, in interviews with those requesting their own deaths, the request should be carefully scrutinized to confirm that the desire is not stemming from financial issues, inadequate care, or out of a self-sacrificial sense of not wanting to be a burden on others; if any of these is suspected, then the patient’s request should not be granted.

Finally, eligibility should be granted to persons with a prognosis of six months or less, and discussion of these matters should begin with a proposal to approve only assisted dying in which the person takes the VAD medication themselves, i.e., self-administration for patients with decision-making capabilities. This is to ensure that VAD in Japan is based solely on the will of the individual. Physician administration, which is dependent upon physician judgment, is prohibited for the time being. We are aware that this very strict bill poses a major problem for patients with incurable neurological diseases, the prognoses of which are estimated to be less than one year, and for patients who cannot take their own medications due to physical problems; these patients would not have access to VAD. However, this extreme limit on eligibility would make it easier for the opposing side to join the discussion table.

The aforementioned Yokohama Regional Court’s clause, i.e., “the patient’s death must be imminent,” [11] was not chosen for application because if a patient’s death is truly imminent, then it is impossible to go through the time-consuming process of confirming the patient’s true intents on multiple occasions. If the patient’s condition is so severe, a calm and collected judgment might not be possible. Patients for whom death is imminent have a shorter time that they will be in suffering relative to those with a six-month prognosis, so it is possible that this judgment might also be permissible.

For the VAD, which has been implemented in Victoria since June 19, 2019, practical problems in the medical field arising from the strictness of the legal procedures and safeguards have also become apparent [24–26]. The prospective approval and oversight process has become bureaucratic. These rigorous procedures take a great deal of time before patients have access to VAD, which is a significant burden for both patients and physicians. The stationary prohibition of physicians initiating discussions with their patients about VAD prevents both the provision of appropriate information as well as informed choice by those who do not have access to or understand medical information on this matter. There is too much paperwork and difficulty in determining patient prognosis and absence of external pressures. It is certainly worth considering the negative impact on the mental health of physicians involved in VADs, and conflict between accepting or refusing requests about a patient’s VAD [24–26]. Our proposal is more conservative and time-consuming and would undoubtedly have more practical problems than in Victoria. However, we would argue that it is important to strike a balance between improving access and safeguarding in the Japanese community. An initial implementation should proceed with great caution. A optimal system will not be easily established. This is a serious social experiment in which human lives may be at stake. We propose this route with the full knowledge that severe and specific problems will emerge in the domestic clinical setting.

We argue that the most important safeguard in Japan is guaranteeing an individual’s voluntariness and agency, as well as preventing any external influence toward choosing death. The discussion on VAD legislation must also begin. Lack of necessity and prematurity are insufficient reasons for snuffing out the discussion. Medical professionals and society are not walking alongside and sharing the feelings of those who carry agony in living. The clean and simple argument that continuing to live is always the best, regardless of the situation, does not address the feelings and experiences of patients living in suffering. We will all face death one day, and a free and open discussion is needed [10].

Finally, as we begin discussion on VAD, there is also an urgent need for Japanese society to establish and enact a basic act for patient rights. The act should include respect for a patient’s right to self-determination, the right to refuse unwanted treatment including not-starting and stopping life-sustaining treatments, the right to prepare legally binding advance directives and the right to decline the preparation of such directives, and access to nationally insured healthcare including quality palliative care. The act should also refer to a healthcare professional’s right to conscientious objection to VAD [28].

## Data Availability

Not applicable because our study did not use any data or materials except published literature.

## Abbreviations

VAD: Voluntary assisted dying
VADA 2017: Voluntary Assisted Dying Act 2017, Victoria
